# Recent Advances in Sodium-Ion Batteries: Cathode Materials

**DOI:** 10.3390/ma16216869

**Published:** 2023-10-26

**Authors:** Thang Phan Nguyen, Il Tae Kim

**Affiliations:** Department of Chemical and Biological Engineering, Gachon University, Seongnam-si 13120, Gyeonggi-do, Republic of Korea; phanthang87@gmail.com

**Keywords:** sodium-ion batteries, cathode materials, inorganic cathodes, organic cathodes, Prussian blue analogs

## Abstract

Emerging energy storage systems have received significant attention along with the development of renewable energy, thereby creating a green energy platform for humans. Lithium-ion batteries (LIBs) are commonly used, such as in smartphones, tablets, earphones, and electric vehicles. However, lithium has certain limitations including safety, cost-effectiveness, and environmental issues. Sodium is believed to be an ideal replacement for lithium owing to its infinite abundance, safety, low cost, environmental friendliness, and energy storage behavior similar to that of lithium. Inhered in the achievement in the development of LIBs, sodium-ion batteries (SIBs) have rapidly evolved to be commercialized. Among the cathode, anode, and electrolyte, the cathode remains a significant challenge for achieving a stable, high-rate, and high-capacity device. In this review, recent advances in the development and optimization of cathode materials, including inorganic, organometallic, and organic materials, are discussed for SIBs. In addition, the challenges and strategies for enhancing the stability and performance of SIBs are highlighted.

## 1. Introduction

**Figure 1:**
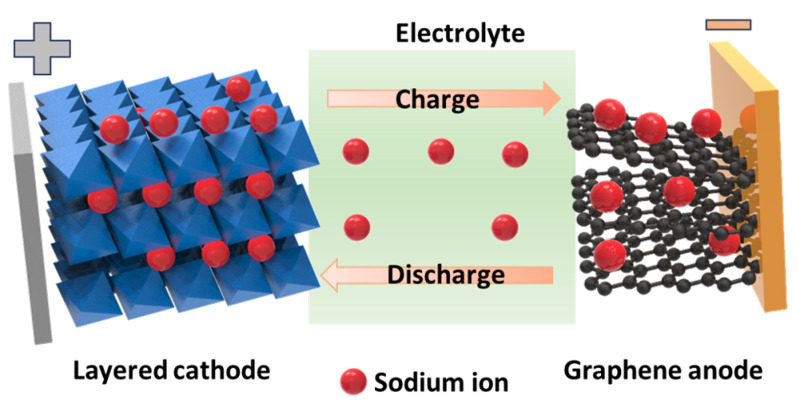
Schematic of the simple operation of a sodium-ion battery employing a layered cathode and graphene anode.

The invention of batteries has played a key role in the development of miniaturized electrical devices. In particular, the use of lithium-ion batteries (LIBs) allows portable devices to continuously operate with no, or rarely occurring, disruptions [1]. LIBs are currently used in smartphones, tablets, notebooks, and vehicles. The significant achievement of LIBs is owing to the strong activity of lithium-ion insertion and desertion in storage materials with a high specific capacity (approximately 3860 mAh g^−1^) [2,3,4,5]. However, with an increase in capacity, various issues associated with LIBs need to be overcome, including safety, toxicity, and cost-effectiveness [6,7,8,9,10]. Meanwhile, sodium is abundantly available on Earth and has similar properties to lithium in storage devices, which is why it is receiving notable attention [11]. The use of sodium-ion batteries (SIBs) reduces the danger of lithium owing to its strong activation; furthermore, the cost and environmental issues can also be resolved [9,12,13,14,15,16,17,18,19,20,21,22]. Considering the development of LIBs, SIBs have become a promising alternative to LIBs. The working mechanisms of LIBs and SIBs are based on the storage of Li and Na ions in two materials with different potentials separated by an electrolyte, as shown in Figure 1. The insertion and desertion of Na ions in the anode and cathode through the electrolyte create and reduce the potential between the two electrodes, corresponding to charge and discharge processes, respectively. Anode materials can also undergo conversion reactions that react with Na ions, forming alloy states that allow high capacities, such as in expanded graphite (284 mAh g^−1^), TiO_2_-based anodes (200–300 mAh g^−1^), antimony sulfides (Sb_2_S_3_) (730 mAh g^−1^), Sn_4_P_3_ (>1100 mAh g^−1^), and phosphorous with a theoretical capacity of ~2596 mAh g^−1^, among others [23,24,25,26,27,28,29,30]. However, the development of a sodium cathode continues to present limitations such as an unstable and low capacity of 100–200 mAh g^−1^. SIB cathode materials include a variety of inorganic compounds (metal oxides, phosphates, pyrophosphates, etc.) and organic or organometallic materials [31,32]. Although achievements have been reported for SIBs and they are being commercialized, the current cathode material has been significantly improved and developed to have better electrochemical properties [33,34,35].

In this review, we provide an overview of the current state of development of SIB cathode materials, including inorganic, organic, and organometallic materials. Recent advances in the development and optimization of these materials have been extensively discussed. In addition, the challenges and strategies related to enhancing the stability and performance of SIBs are highlighted.

## 2. Review of SIB Cathode Materials

### 2.1. Inorganic Compounds

#### 2.1.1. Layered Oxide Materials (Na_x_MO_2_)

**Figure 2:**
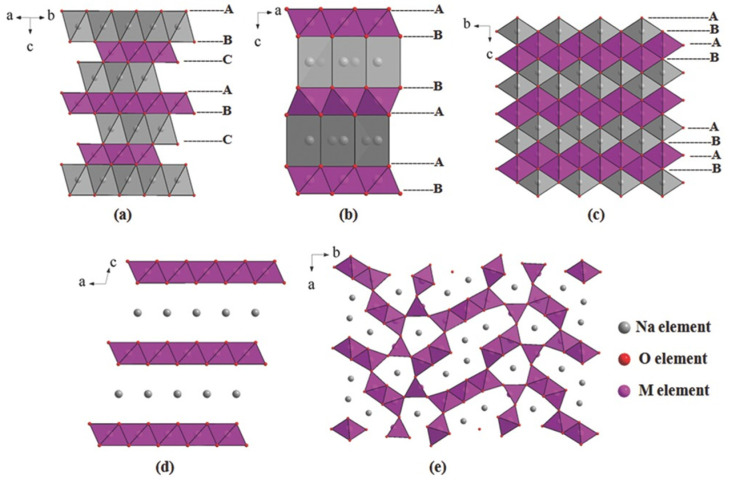
Layered metal oxide: (**a**) O3-phase, (**b**) P2-phase, (**c**) O2-phase, (**d**) birnessite-type layered oxides and (**e**) tunnel oxides. The letters A, B, C are packing patterns of oxygen ions in an abc coordinate system. Reproduced from ref. [37]; Copyright 2015 Wiley-CH.

The layered oxide materials used for SIBs mostly consist of transition-metal oxides [36]. There are two common phases of NaMO_2_, which are the O3 and P2 phases, classified based on the different stacking of the oxygen ion frameworks as ABCABCABC (O3) or ABBAABBA (P2), as shown in Figure 2a,b [37,38]. In addition, the O2 phase and birnessite are the layered structures with the tightest and loosest packing, respectively, as shown in Figure 2c,d [39,40,41]. Among these phases, O3 phase can provide a high Na content and high specific capacity, which enables its application in full cells. However, the degradation of structure during cycling limits its application. To maintain structure, foreign metals with a large ionic radius such as Fe, Cr, Ti, and V can be used introduced [42]. On the other hand, P2 phase has a lower Na content but a wider layer spacing, which leads to faster diffusion of Na^+^ ions and improves structural stability during cycling. Similar to LIBs, compounds of Na with Co, Ni, and Mn oxides have layered structures, such as Na_x_CoO_2_, Na_x_NiO_2_, and Na_x_MnO_2_ [43,44,45,46]. However, owing to the large size of Na ions, the behavior of CoO_6_ or NiO_6_ in the lattice with the intercalation of Na varies from that of Li [47]. Na_x_CoO_2_ and Na_x_NiO_2_ compounds have exhibited low capacities below or near 100 mAh g^−1^ [48,49]. Reddy et al. fabricated P2-Na_x_CoO_2_ using the sol–gel method, capable of delivering a capacity of approximately 121 mAh g^−1^ at a rate of 0.1 C [50]. Similarly, NaNiO_2_ exhibits a capacity of only approximately 80 mAh g^−1^ [51]. Meanwhile, Na_x_MnO_2_ is a more promising cathode material owing to the multiple oxidation states of the Mn ions in the zigzag layers of the edge-sharing MnO_6_; therefore, this cathode exhibits a high theoretical capacity of approximately 240 mAh g^−1^ [52,53,54]. Na_x_MnO_2_ can be synthesized from either NaOH and Mn salt or MnO_2_. Ma et al. used monoclinic NaMnO_2_ as a cathode for SIBs and demonstrated a high first discharge capacity of approximately 185 mAh g^−1^ in the 2–3.8 V range [55]. Billaud et al. synthesized β-NaMnO_2_ which achieved a high capacity of approximately 190 mAh g^−1^ and retained a capacity of 100 mAh g^−1^ after 100 cycles at 2 C [56]. Kubota et al. investigated the effect of the voltage change on distorted O3-phase (O’3) NaMnO_2_ and found that a phase transition of NaMnO_2_ occurs above 3.52 V, leading to a decrease in crystallinity, thereby rapidly degrading the capacity during the cycling test [57].

**Figure 3:**
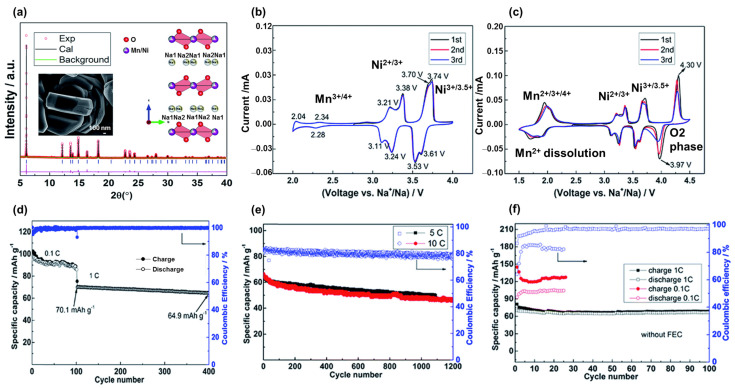
(**a**) X-ray diffraction pattern and inset scanning electron microscopy image of the Na_2/3_Ni_1/3_Mn_2/3_O_2_ material. Cyclic voltammograms (CVs) at (**b**) 2.0–4.0 V and (**c**) 1.5–4.5 V. Cycling performances at (**d**) 0.1 C and 1 C for 400 cycles, (**e**) 5 C and 10 C for 1200 cycles, and (**f**) of the electrolyte without the additive (FEC). Reproduced with permission from ref. [67]; Copyright 2019 Royal Chemical Society.

The drawback of layered materials is their unstable structure in air storage and during cycling; therefore, their capacities can be rapidly or irreversibly degraded [58,59]. Due to its hygroscopic nature, NaMO_2_ is unstable in air and in moist environments; therefore, its applications are limited. To improve the performance of NaMnO_2_, the partial replacement of Mn with other metals, such as Li, Ni, Co, Al, Fe, and Zn, has been investigated [60,61,62,63,64,65,66]. Kwon et al. proposed the use of a P2-NaLiMnO_2_ cathode material that exhibited a high reversible capacity of approximately 160 mAh g^−1^ [60]. The insertion of Li ions as dopants led to an inhomogeneous electrostatic repulsion between the Mn and Na ions, thereby enhancing the stability of β-Na_0.7_[Mn_1-x_Li_x_]O_2+y_, which exhibited a stable cycling capacity for over 120 cycles without a faded capacity. Liu et al. investigated the use of P2-Na_2/3_Ni_1/3_Mn_2/3_O_2_ as a cathode material for SIBs simply synthesized via a novel sol–gel method (NSG) by employing polystyrene as an additive, as shown in Figure 3a [67]. The main active metal is Ni with Ni^2+^/Ni^4+^ states that contribute to the redox-pair peaks at a voltage between 3.0–4.0 V and a minor Mn^3+^/Mn^4+^ redox potential between at 2.0–3.0 V as shown in Figure 3b. Meanwhile, Mn^4+^ effectively maintains the structure of NaNiMnO_2_, thereby significantly improving its stability. At voltages below 2.0 V, the Mn^4+^ ions were activated and reduced to Mn^3+^, suffered a disproportional reaction, and dispersed into the electrolyte (Mn^3+^ solid → Mn^4+^ solid → Mn^2+^ electrolyte), and the redox at ~4.0–4.5 V was related to the phase transition from P2 to O2 phase due to the stacking faults, as shown in Figure 3c. Therefore, the material can be rapidly degraded below 2 V. The NSG Na_2/3_Ni_1/3_Mn_2/3_O_2_ cathode exhibited a reversible capacity of approximately 100 mAh g^−1^ and an excellent rate performance even at rates of 5 C and 10 C, as shown in Figure 3d–f. Nanthagopal et al. used NaFe_0.5_Mn_0.5_O_2_ as a cost-effective SIB cathode material which exhibited a specific capacity of approximately 170 mAh g^−1^ and retained a capacity of approximately 114 mAh g^−1^ after 100 cycles [61]. Liu et al. doped Al ions into NaMnO_2_ to form P2-Na_0.67_Al_0.1_Mn_0.9_O_2_ as a SIB cathode material [62]. The strong bonding of Al–O leads to enhanced Na spacing; therefore, Na ions can easily insert and desert into the cathode material. Hence, the presence of Al also reduces the Jahn–Teller effect of the phase transition between P2-P2′, which could cause structural defects and collapse during cycling [68]. Therefore, P2-NaAl_0.1_Mn_0.9_O_2_ can deliver a high capacity of 175 mAh g^−1^ with high stability and rate performance. Replacement with metals such as Ni, Co, Al, and Fe with higher redox states increases the average oxidation state of Mn ions (>3+), which mitigates the structural deterioration resulting from the Jahn–Teller effect and partially increases the redox potential [69]. For example, the redox potential of Mn^3+/4+^ is below 3.0 V, and the partial reduction of Mn^3+^ to Mn^2+^ leads to the dissolution of Mn^2+^ into electrolyte, resulting in structural degradation and reduced capacity. Introducing Fe ions into the structure causes a Fe^3+/4+^ redox between 3.0–4.0 V, increasing the average oxidation state of Mn ions and improving the stability. The higher redox potential of Fe^3+/4+^ also contributes to the working potential of the cathode material. Moreover, Mn and Fe are Earth-abundant elements that promise low-cost and environmentally friendly production. Similar to lithium-based layered metal oxides, the O3 phase of NaTMnO_x_ (T = Ni, Co, Fe) has a high sodium content and provides a more stable layered oxide, making it applicable to full cells [70,71].

A combination of more than three metals was also investigated, including NaLiNiMnCoO_2_, NaLiNiMnO_2_, NaFeMnTiVO_2_, and NaMnNiCuMgTiO_2_ [72,73,74,75]. Kataoka et al. prepared a multi-metal complex of NaLiNiMnCoO_2_ via co-precipitation and electrochemical ion-exchange methods [72]. The produced Na_0.95_Li_0.15_(Ni_0.15_Mn_0.55_Co)O_2_ was then employed as a highly stable cathode which delivered a capacity of greater than 200 mAh g^−1^ for over 40 cycles. Xu et al. investigated the effect of Li ions on NaLiNiMnO_2_ cathodes in SIBs and determined the importance of each element as follows [74]: The Ni metal was fully oxidized to Ni^4+^ to balance the overall charge of the cell, which also prevented the Jahn–Teller distortion owing to the active Mn^3+^. Moreover, Ni ions also contributed to the high-voltage redox state of the cathode, widening the range of the working potential from 2.0 to 4.4 V. Li ions were found surrounding Ni^4+^ through NMR resonance methods, which indicated that Li could easily migrate to this material. The remaining Li during cycling enhanced the capacity retention; therefore, this cathode delivered a high reversible capacity of 140 mAh g^−1^ in the 2.0–4.4 V range. Other elements have also been doped to improve the performance of Mn-based cathodes, such as boron-doped NaLiNiFeMnO_2_, Y-doped P2-type NaNiMnO_2_, and Mg-doped NaMnMgO_2_ [76,77,78].

In addition to Co-, Ni-, and Mn-based metal oxide cathodes, Cr-, Cu-, and Fe-based oxides have also received significant attention [79,80,81,82,83,84]. Yu et al. developed carbon-coated NaCrO_2_ as a SIB cathode via an emulsion-drying method that exhibited an excellent performance at a high rate of 50 C with a capacity of approximately 100 mAh g^−1^ [79]. The NaCrO_2_ cathode also demonstrated significant thermal stability up to 400 °C. At temperatures above 290 °C, instead of oxygen evolution owing to the thermal decomposition, NaCrO2 decomposed to Na_0.5_CrO_2_ and CrO_2_ phases. Moreover, Na_0.5_CrO_2_ continued to exhibit a stable layered structure from the insertion and desertion of the Na ions. Na_x_CuO_2_ and Na_x_FeO_2_ also have layered structures and deliver a capacity of approximately 100–200 mAh g^−1^ [80,81,85,86]. Lee et al. found that Fe^3+^/Fe^4+^ in Na_x_FeO_2_ was unstable during the redox process, leading to the formation of an octahedral structure, preventing the diffusion of Na ions and degrading the capacity [86]. A typical issue in layered metal oxide materials is the collapse of the structure during the insertion and desertion of sodium ions [37].

#### 2.1.2. Tunnel Oxides

**Figure 4:**
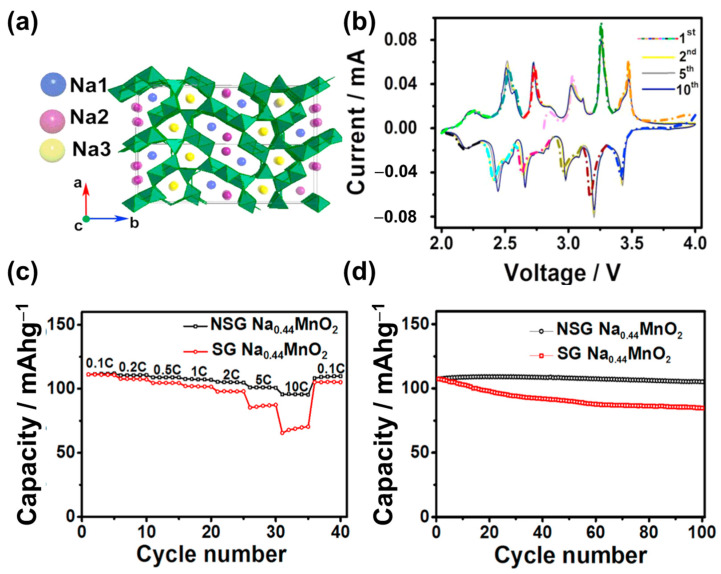
(**a**) Illustration of orthorhombic Na_0.44_MnO_2_, (**b**) CV of (novel sol–gel-synthesized) NSG-Na_0.44_MnO_2_, (**c**) rate performance at current rates between 0.1–10 C, and (**d**) cycling performances of NSG-Na_0.44_MnO_2_ and (conventional sol–gel-synthesized) SG-Na_0.44_MnO_2_ at 0.5 C. Reproduced with permission from ref. [98]; Copyright 2016 Elsevier.

The Na_x_MO_2_ tunnel oxide consists of M^4+^ and M^3+^ ions at the MO_6_ and MO_5_ sites, respectively, as illustrated in Figure 2e [87,88,89,90]. The mixing of MO_6_ and MO_5_ creates a tunnel structure that allows Na^+^ ions to easily diffuse along the tunnels. This structure was first discovered by Parant et al. (1971) for Na_x_MnO_2_ (x < 1) [91]. It is worth noting that this structure was simply synthesized using various approaches, such as sol–gel, hydrothermal, spray pyrolysis, and microwave-assisted methods [92,93,94,95]. Na_0.44_MnO_2_ is the most noteworthy tunnel oxide owing to its large tunnels, high theoretical capacity of approximately 121 mAh g^−1^, and high stability [96,97]. He et al. used a polymer-pyrolysis method to fabricate Na_0.44_MnO_2_ nanoplates, which exhibited an outstanding capacity of approximately 96 mAh g^−1^ at a rate of 10 C [98]. However, the capacity of this material could not be improved owing to the fully charged and discharged states of the Na_0.22_MnO_2_ and Na_0.66_MnO_2_ phases, respectively [96]. Therefore, methods were developed to solve this problem, including cation/anion substitution and surface coating. In cation substitution, Mn^4+^ can be replaced by Ti, Fe, or Zr or by the partial replacement of Na with Li ions [99,100]. Shi et al. doped Zr ions in Na_0.44_MNO_2_ as a high-performance SIB cathode, which exhibited a high capacity of approximately 117 mAh g^−1^; at a high rate of 5 C, the capacity was reversible at approximately 97 mAh g^−1^, as shown in Figure 4 [101]. Defects at the Na, MnO_6_, and MnO_5_ sites create Na1 and Na2 in the S-shaped tunnels and Na3 in the smaller pentagonal tunnel, which allows the insertion and desertion of Na, thereby increasing the cycling performance, as illustrated in Figure 4a. As shown in Figure 4b, the insertion and desertion of the Na ions demonstrate six pairs of redox peaks with a small gap between each peak, allowing the rapid diffusion of Na ions. Therefore, the current rate and cycling performances were excellent at 10 C for over 100 cycles, as shown in Figure 4c,d. Zheng et al. used the composition of layered Na_2_Mn_3_O_7_ and Na_0.44_MnO_2_ as a SIB cathode, which delivered a high specific capacity of approximately 135 mAh g^−1^ and retained a capacity of 88% of the initial state after 100 cycles at 0.2 A g^−1^ [102]. Further improvement remains a challenge for scientists because of the change in structure owing to doping or the substitution of ions such as Co and Al [96]. Zhou et al. used Co-substituted Na_0.44_Mn_1-x_Co_x_O_2_ and found that the structure of the tunnel oxide changed to a layered structure [103]. The substitution of Al can form a mixture of the tunnel and layered phases in NaAl_0.1_Mn_0.9_O_2_ SIB cathodes [104]. In comparison to layered oxides, the tunnel oxides provide large channels for diffusion of Na ions, improving conductivity and stability. However, the low content of Na ions in the structure results in low specific capacity (~100 mAh g^−1^) and energy density. These drawbacks limit commercialization.

#### 2.1.3. Polyanionic Compounds

##### Phosphate-Based Compound

Polyanionic compounds are generally constructed by a tetrahedral XO_4_ group with Na and Me (Fe, V, Co, or Mn) or MeO_x_ [105,106,107,108]. Basically, olivine NaFePO_4_ consists of tetrahedral PO_4_ and octahedral FeO_6_ sites, forming a framework that holds Na ions in the lattice or allows the diffusion of Na ions [109]. NaFePO_4_ is a cost-effective material owing to its abundance of elements and high theoretical capacity of approximately 154 mAh g^−1^. NaFePO_4_ exists in two phases: maricite and olivine. The maricite phase is a stable structure with cavities that trap Na ions, preventing their diffusion [105,110]. Meanwhile, the less stable olivine phase has a one-dimensional channel, allowing the diffusion of Na ions through this pathway. Therefore, the olivine phase is more attractive, and improving the stability of this structure with various types of doping has also been investigated [58]. Wang et al. used the DFT simulation method to predict the effect of doping Li into NaFePO_4_ in both maricite and olivine phases [110]. The results demonstrated that when the Li:Na ratio was above 25%, the olivine phase was more stable than maricite, whereas the presence of Li destabilized the maricite structure. Ali et al. synthesized olivine NaFePO_4_ via an ion-exchange method from LiFePO_4_ for SIBs, which was then wrapped with polythiophene (PTh) to enhance the stability of the material [111]. NaFePO_4_ with PTh experiences the expansion of the insertion and restoration of its structure, which is indicated by the minuscule difference in the unit cell volume (from 320.6 to 320.3 Å^3^). Therefore, the NaFePO_4_/PTh cathode exhibits a high reversible capacity of approximately 142 mAh g^−1^. Altundag et al. used an electrochemical process to exchange Li with Na from LiFePO_4_ to NaFePO_4_, which delivered a capacity of approximately 74 mAh g^−1^. Olivine NaFePO_4_ structures are promising for SIBs; however, their fabrication remains a major obstacle that requires further investigation. Maricite NaCoPO_4_ (red phase) offered a high redox potential of Co^2+/3+^ at 4.0–4.6 V; however, the reversible capacity in a SIB was low at ~35 mAh g^−1^ [107]. Similarly, for NaMnPO_4_, it was also reported that the specific capacity of maricite phase exhibited a low capacity of ~40 mAh g^−1^ [108]. Priyanka et al. reported a different precursor (Mn) effect for the quality of NaMnPO_4_. A cathode prepared with a precursor from manganese acetate showed outstanding performance with an initial capacity over 100 mAh g^−1^ [112]. The high performance resulting from the acetate-based Mn precursor could be due to the decomposition of acetate creating a carbon source to enhance the conductivity and porosity of NaMnPO_4_. Venkatachalam et al. synthesized maricite NaMnPO_4_ with poly-ethylene glycol (PEG) and diethylene glycol (DEG), which can increase the specific capacity from 50 to ~100 mAh g^−1^ at 0.1 C [113]. The high contribution of carbon sources from PEG and DEG significantly enhanced the conductivity and protected the structure of this material. Phosphate compounds possess a high thermal stability (~600 °C); however, their major drawbacks are low electronic conductivity and low specific capacity, limiting their application in full-cell SIBs [114].

##### NASICON

**Figure 5:**
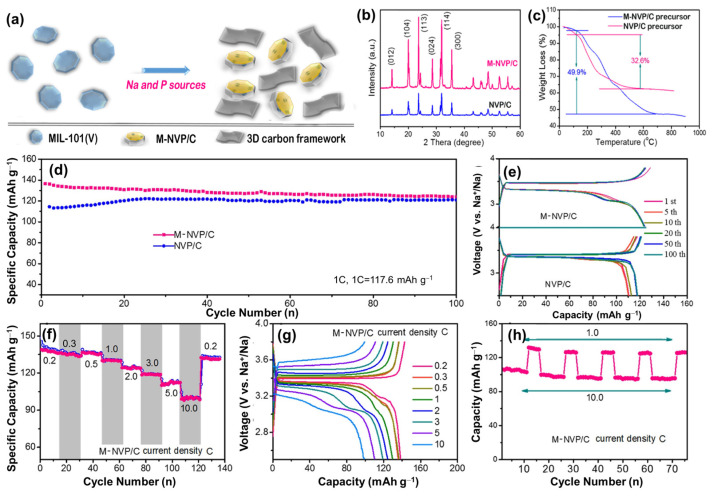
(**a**) M-NVP/C synthesis scheme, (**b**) XRD, (**c**) thermogravimetric analysis plots, (**d**) cycling performance at 1 C, (**e**) voltage profiles of M-NVP and NVP/C; (**f**) rate performance, (**g**) voltage profiles at different C rates ranging from 0.2 C–10 C, and (**h**) rate performance at 1 C and 10 C of the M-NVP cathode. Reproduced with permission from ref. [124]; Copyright 2019 Elsevier.

A Na super-ionic conductor (NASICON) can be used as an electrolyte and electrode material owing to its 3D-open framework of Na_x_M_2_(PO_4_)_3_ (M = V, Fe, Ti, Nb, Zr) [115]. NASICON comprises MO_6_ and PO_4_ polyhedral sites in a framework that creates large channels for Na diffusion. This structure was first proposed by Hong and Goodenough in a Na_1+x_Zr_2_P_3-x_Si_x_O_12_ compound (P can be replaced by Si, S, Mo, and As) [116,117]. Owing to its high stability, high Na conductivity, and wide electrochemical windows (1.85–4.9 V vs. Na/Na^+^), NASICON is also applied as a solid electrolyte in SIBs [118]. The ion exchange of Zr^4+^ with Li^+^, K^+^, and Ag^+^ was first performed, while Si^4+^ was stabilized in the structure. As a complete NASICON with three full Na ions, Na_3_V_2_(PO_4_)_3_ (NVP) quickly received significant attention as a promising candidate material for providing a high probability of sodium insertion and desertion [119,120]. NVP has a theoretical capacity of ~117.6 mAh g^−1^ and a high redox voltage range of 3.3–3.4 V [121]. Therefore, with the modification process including the addition of conductive carbonaceous materials, NVP conductivity can be enhanced, exhibiting a notable rate performance [122]. Song et al. used a carbothermal reduction method to fabricate NVP, which exhibited a high capacity of approximately 117 mAh g^−1^ [123]. Cao et al. synthesized high-crystalline M-NVP/C nanoparticles using MIL-101 as the V source combined with an amorphous carbon layer, as shown in Figure 5a–c [124]. The M-NVP/C cathode delivered a high capacity of approximately 136 mAh g^−1^ and an excellent performance at high current rates of 1 C, 5 C, and 10 C for 1000 cycles, as shown in Figure 5d–h. In addition, the substitutions of Na, V, and P were found to be effective in improving its stability [125,126,127]. Lim et al. used the sol–gel method to produce Na_3−x_K_x_V_2_(PO_4_)_3_/C [125]. The use of K ions helped increase the Na-ion diffusion pathway and improved the stability and rate performance of NVP. Meanwhile, Mg replaced the V ions and improved the conductivity of the material; Mg_0.07_-NVP delivered a reversible capacity of approximately 113 mAh g^−1^ at 0.1 C and a high reversible capacity of 95 mAh g^−1^ at 10 C [127]. Pal et al. used Si-doped NVP as a replacement for P to form Na_3.1_V_2_(PO_4_)_2.9_(SiO_4_)_0.1_, which delivered an initial capacity of approximately 112 mAh g^−1^ and enhanced the capacity in comparison to NVP at high rates [126]. Fluorine is also favorable for substitution in the PO_4_ group owing to the larger ionicity of the metal when bonded with F compared to O. Moreover, F atoms were found to be compatible with the PO_4_ group in polyanionic compounds; therefore, the fluorine phosphate compound is believed to enhance the diffusion of Na^+^ ions [128]. Song et al. fabricated Na_3_V_2_(PO_4_)_2_F_3_ via a carbothermal reduction method as a SIB cathode material [129]. The existence of F in NVP changes the electrochemical behavior from a single redox couple at 3.06/3.72 V to a double redox couple at 3.24/3.91 and 3.83/4.26 V; therefore, the specific power density is improved.

#### 2.1.4. Pyrophosphates

Pyrophosphate Na_x_MP_2_O_7_ consists of MO_6_ (M = V, Fe, Mn, Co, Ni) sites and a P_2_O_7_ group (interconnected PO_4_–PO_4_) that forms a framework with Na ions [130,131,132,133,134,135]. This framework allows the diffusion of Na ions; therefore, it is also a stable cathode material for SIBs. Barpanda et al. revealed that Na_2_FeP_2_O_7_ was constructed by corner-sharing FeO_6_–FeO_6_ to form Fe_2_O_11_, which combines with the P_2_O_7_ group to form a triclinic structure [136]. After calcination at temperatures above 560 °C, the triclinic Na_2_PeP_2_O_7_ transformed into a monoclinic phase, which improved the stability of this material during cycling. Kim et al. used the defect engineering of Na in Na_2_CoP_2_O_7_ to produce a high-voltage cathode for SIBs [137]. The deficiency of the Na-stabilized structure of Na_2-x_CoP_2_O_7_ (x > 0.2) was also found in Fe, Ni, and Mg pyrophosphates, such as Na_1.66_Fe_1.17_P_2_O_7_, Na_1.82_Ni_1.09_P_2_O_7_, and Na_1.82_Mg_1.09_P_2_O_7_ [138,139,140,141]. Specifically, Na_2-x_Co_2_P_2_O_7_ (x > 0.2) achieved a high average voltage of approximately 4.3 V versus Na/Na^+^ with a specific capacity of approximately 80 mAh g^−1^. Owing to the similar roles of the V, Fe, Mn, Co, and Ni transition metals in the structure, the replacement of a cheaper metal such as Fe and the improvement of the voltage by using Co and Ni in other pyrophosphate materials were investigated. Liu et al. investigated the use of Na_2_Mn_3-x_Fex(P_2_O_7_)_2_ as a SIB cathode and revealed that the diffusion coefficient of Na ions improved by two orders of magnitude with x > 0.5 [142]; however, the capacity remained low at approximately 86 mAh g^−1^. Deng et al. fabricated a high-Na-content Na_7_V_3_(P_2_O_7_)_4_, which was also used as a high-voltage SIB cathode at an average voltage of approximately 4.0 V with a capacity of approximately 80 mAh g^−1^. Kumar et al. proposed the use of Mo_2_P_2_O_11_ as a SIB cathode with a high capacity of approximately 90 mAh g^−1^ [143]. The structure of Mo_2_P_2_O_11_ is also a framework of MoO_6_ sites with PO_4_ and P_2_O_7_ sites. Therefore, incomplete or shared atoms can form a tunnel phase in phosphates. Pyrophosphate-based cathode materials remain limited owing to their low capacity because the P_2_O_7_ group is large; therefore, the replacement of this group with PO_4_ and F could be an efficient method to enhance their capacity and stability [131,144,145]. Pu et al. fabricated a Na_4_Fe_3_(PO_4_)_2_P_2_O_7_/C nanosphere for SIB cathodes, which delivered a high capacity of approximately 128 mAh g^−1^ at 0.2 C and a remarkable rate performance at 100 C with a capacity of >70 mAh g^−1^ [144]. Kundu et al. used fluorine to modify pyrophosphates and produced Na_4_NiP_2_O_7_F_2_ as a high-voltage cathode for SIBs [145]. The presence of strong electronegative F^−^ groups incorporated with P–O moieties increased the redox of Ni^2+^/Ni^4+^ owing to the high-charge region between 4.7 and 5.2 V. However, this material was not sufficiently stable to retain the capacity of the SIBs.

#### 2.1.5. Silicates

**Figure 6:**
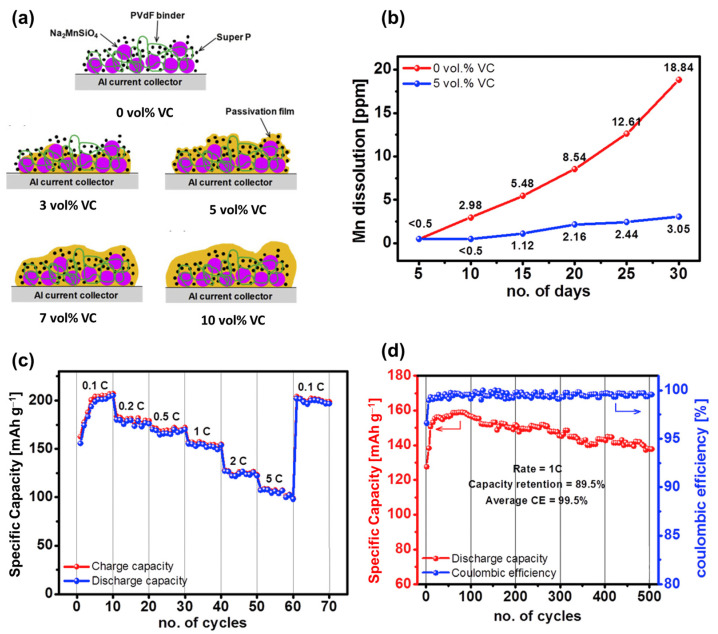
(**a**) Illustration of the passivated layer on the Na_2_MnSiO_4_ cathode with additive vinylene carbonate (VC). (**b**) Mn dissolution of the Na_2_MnSiO_4_ electrode in electrolytes at room temperature. (**c**) Rate and (**d**) cycling performances of Na_2_MnSiO_4_ with 5 vol% VC at 1 C. Reproduced from ref. [157]; Copyright 2017 Elsevier.

Silicate compounds, such as lithium orthosilicate Li_2_FeSiO_4_ with a theoretical capacity of approximately 300 mAh g^−1^, generally have a higher theoretical capacity than other polyanions owing to their low molecular weight [146]. Similar to Li_2_FeSiO_4_, the sodium silicate Na_2_MSiO_4_ compound consists of MO_4_ (M = Fe, Ni, Mn, Co) and SiO_4_ sites, forming a framework that allows the diffusion of Na ions [147,148,149]. Silicates were previously popular in the glass industry owing to their high thermal and physical stabilities [150]. Co/Fe-compound sodium silicates were predicted to exhibit anti-site-exchange behavior, promising to be stable electrode materials [151,152]. Na_2_FeSiO_4_ is the most promising silicate compound, having a high theoretical capacity of approximately 276 mAh g^−1^ [153]. Kee et al. fabricated Na_2_FeSiO_4_ using a solvothermal method as a SIB cathode material, which exhibited a high initial capacity of approximately 126 mAh g^−1^ [154]. However, the material’s capacity quickly degrades owing to the collapse of the crystal into an amorphous structure. Meanwhile, Na_2_CoSiO_4_ demonstrated a better stability; however, its capacity was approximately 100–120 mAh g^−1^ [148,149,155]. Guan et al. resolved the stability issue of Na_2_FeSiO_4_ by introducing a fluorine dopant, which delivered a high capacity of approximately 270 mAh g^−1^ as a SIB cathode [156]. The presence of fluorine with strong electronegativity reduced the strain of the Na-hosting cathode, and the volume change during the charge and discharge process was only approximately 1.38%. Law et al. prepared Na_2_MnSiO_4_ that delivered a high capacity of approximately 210 mAh g^−1^ at 0.1 C and a reversible capacity of ~100 mAh g^−1^ at 5 C [157]. The excellent performance of Na_2_MnSiO_4_ was achieved owing to the additional vinylene carbonate in the electrolyte, which allowed the formation of a passivated layer during cycling, as shown in Figure 6. Therefore, the drawback of an unstable structure can be resolved by using a protective layer or surface passivation.

### 2.2. Organic Compounds

**Figure 7:**
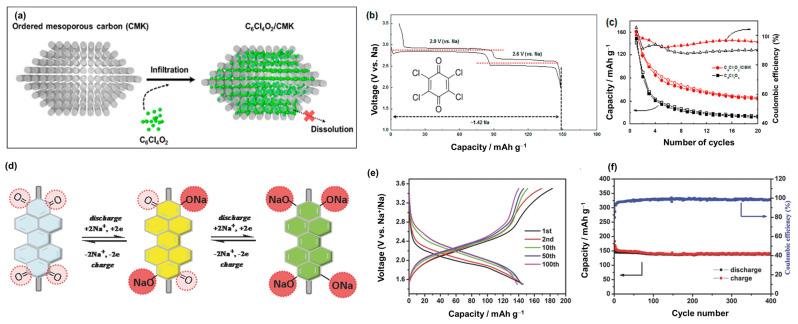
(**a**) C_6_Cl_4_O_2_/CMK synthesis scheme, (**b**) voltage profiles of C_6_Cl_4_O_2_ at 10 mA g^−1^, and (**c**) cycling performance of C_6_Cl_4_O_2_ with and without CMK. Reproduced with permission from ref. [161]; Copyright 2015 American Chemical Society. (**d**) Schematic diagram of the redox mechanism, (**e**) voltage profiles, and (**f**) cycling performance of PTCDA-based PIs at 0.1 C. Reproduced with permission from ref. [159]; Copyright 2014 Wiley-CH.

The development of flexible devices and environmentally friendly materials has encouraged the application of organic compounds as cathode materials in energy storage systems, such as LIBs and SIBs [158]. Ranging from small molecules to high-molecular polymers, organic materials are promising for applications in green renewable energy in the future. For example, the molecular structure of Na_4_C_8_H_2_O_6_ (2,5-dihydroxyterephthalic acid, NaDTA) was investigated as a SIB cathode material at working potential windows of approximately 1.6–2.8 V versus Na/Na^+^ and delivered a high capacity of approximately 180 mAh g^−1^ [159]. NaDTA can also be used as an anode material with a capacity greater than 200 mAh g^−1^ owing to it binding up to six Na ions [160]. Kim et al. demonstrated the use of C_6_Cl_4_O_2_ (tetrachloro-1,4-benzoquinone) in a porous carbon template as a cathode of SIBs, as illustrated in Figure 7a,b [161]. The carbon skeleton-supported C_6_Cl_4_O_2_ cathode exhibited a high initial capacity of approximately 160 mAh g^−1^ and an average voltage of approximately 2.72 V, as shown in Figure 7c. Wang et al. produced a polymer from perylene 3,4,9,10-tetracarboxylic dianhydride, pyromellitic dianhydride (PMDA), and 1,4,5,8-naphthalenetetracarboxylic dianhydride, which contained C=O bindings, providing interactions with Na^+^ ions as a cathode for SIBs, as illustrated in Figure 7d [159]. This polymer demonstrated a high reversible capacity of approximately 150 mAh g^−1^ at a working voltage of 1.5–3.5 V and a long lifetime of over 5000 cycles, retaining 87.5% of the capacity in comparison to the initial cycle, as shown in Figure 7e,f. Shen et al. fabricated poly(diphenylaminesulfonic acid sodium) as a SIB cathode material, which delivered a reversible capacity of approximately 100 mAh g^−1^ at a high potential of 3.6 V versus Na/Na^+^ [162]. Wang et al. proposed an extended π-conjugated structure in sodium 4,4′-stilbene-dicarboxylate (SSDC) to provide an increased charge transport, which could easily enhance the rate performance of this organic cathode material in SIB by up to 10 A g^−1^ [163]. Organic cathodes can also be based on the insertion of anions such as ClO_4_^−^. Han et al. employed non-crystalline oligopyrene as a SIB cathode material, which is based on the insertion of ClO_4_^−^ [164]. During cycling, pyrene was oxidized and reduced, allowing the interaction with charged ClO_4_^−^; therefore, each pyrene unit could store ClO_4_^−^ anions. As a SIB cathode, oligopyrene exhibits a high theoretical reversible capacity of approximately 134 mAh g^−1^. Sakaushi et al. fabricated a dicyanobenzene-based aromatic porous honeycomb (APH) cathode with two types of storage mechanisms [165]. APH can store Na+ ions at voltages below 2.8 V. When the voltage is higher than 2.8 V, the APH cathode exhibits a p-doped region, which allows the insertion of ClO^4−^ anions [165]. Therefore, APH has a wide working potential of 1.3–4.1 V versus Na/Na^+^ and a high reversible capacity greater than 120 mAh g^−1^ at 0.1 A g^−1^. Due to the electronic insulative nature of organic materials, they show a low conductivity, limiting their high rate performance. To overcome this limitation, cathode materials should contain high amount of carbon materials (30–60 wt%) to achieve a high rate performance [159,160,162,163,165].

### 2.3. Metal–Organic Compounds: Prussian Blue Analogs

**Figure 8:**
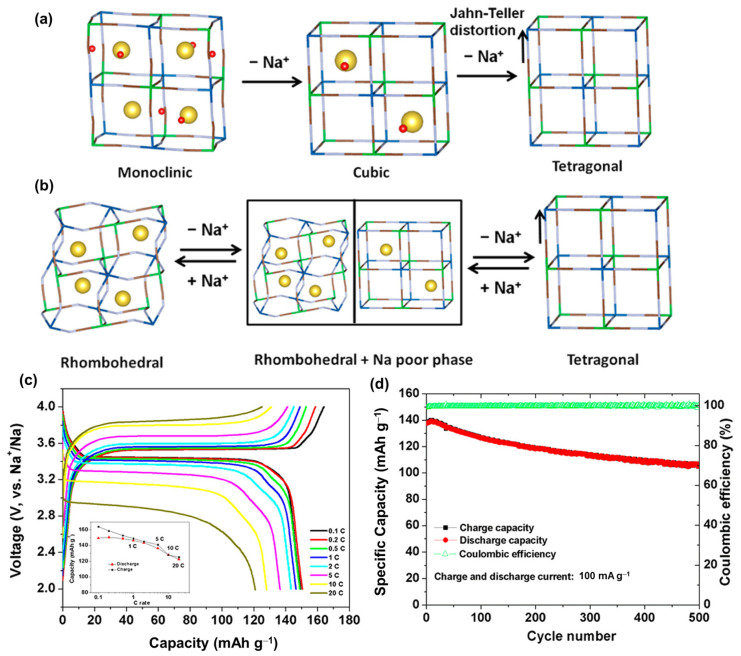
(**a**) Schematic of the monoclinic, cubic M-PBA and (**b**) rhombohedral M-PBA with the insertion and desertion of Na^+^ ions. (**c**) Voltage profiles at different rates from 0.1–20 C and the (**d**) cycling performance of the rhombohedral M-PBA. Reproduced with permission from ref. [183]; Copyright 2015 American Chemical Society.

The combination of inorganic and organic structures has received considerable attention owing to the advantages of both material types [166]. Inorganic materials have a stable structure and high conductivity, whereas organic materials are eco-friendly, easy to process, and safe to use. Recently, the development of organometallic materials in framework structures has introduced an advanced technique for material design, enabling the discovery of new composite properties for metals and organics. Metal–organic frameworks (MOF) can form a tremendous structure from various metal–organic compounds, providing large channels that allow the capture of ions or molecules; therefore, they have been used in various applications, including drug delivery, catalysis, and energy storage [167,168,169]. Simple and famous MOFs used for energy storage are Prussian blue analogs (PBAs), which are alkaline metal ferrocyanides A_x_MFe(CN)_6_ (A = Na, K; M = Fe, Mn, Co, Ni, Cu) [170]. The CN, Fe, and M matrices create a cage-like structure, holding the Na and K ions. PBAs generally exhibit a face-centered cubic structure (Fm3-m) [171,172,173]. The performance of PBAs in SIBs is based on the redox reactions of Fe^2+^/Fe^3+^ and the metal M, believed to have a high theoretical capacity of approximately 170 mAh g^−1^ for SIBs [174]. The basic PBA, which is Na_4_Fe(CN)_6_, contains the highest number of Na ions; however, it is a soluble compound that is easily degraded during cycling [175,176]. Therefore, Yang et al. demonstrated a solid solution of Na_4_Fe(CN)_6_/NaCl in a SIB that exhibited a capacity of approximately 75 mAh g^−1^ [177]. Qian et al. prepared a composition of Na_4_Fe(CN)_6_ with carbon, which also functioned as a SIB cathode [175]. By replacing Na ions with transition metals, the PBA structures were stabilized and widely used in SIBs [178]. Sun et al. used Fe^3+^ to form Fe_4_[Fe(CN)_6_]_3_ for a SIB cathode, which delivered a high capacity of approximately 146 mAh g^−1^ at 20 mA g^−1^ [179]. However, the rate performance and stability remained low; therefore, various types of transition metals, such as Ni, Cu, Mn, and V, have been used to create more stable and high-conductivity cathodes [180,181,182]. Song et al. fabricated Na_2_MnFe(CN)_6_ (Mn-PBA) and found that the removal of water molecules from the material significantly improved its performance, as shown in Figure 7 [183]. Mn-PBA and other PBAs naturally have monoclinic (Na-rich) or cubic structures that are partially supported by water molecules in their crystals. After removing the water molecules, Mn-PBA changed into a distorted framework or rhombohedral structure. Owing to the irreversibility of the monoclinic phase to the cubic phase, the Na per unit was reduced. Hence, the Jahn–Teller distortion of Mn^3+^ led to the degradation of the structure, thereby reducing the capacity, as illustrated in Figure 8a. It is worth noting that the rhombohedral phase allows the Na^+^ ions to be captured more efficiently; therefore, the water-removed Mn-PBA cathode enhanced the electrochemical performance, delivering a high capacity of approximately 150 mAh g^−1^ and a high rate performance even at 20 C, as shown in Figure 8b–d. However, dried Mn-PBA quickly absorbs water molecules; therefore, its fabrication and application remain limited. Hu et al. investigated the effect of Ni replacement in Mn-PBA as a SIB cathode [184]. Ni with a 10% replacement of Mn in Mn-PBA can enhance stability, delivering a capacity of approximately 110 mAh g^−1^ and a high rate performance. Xu et al. proposed a scalable preparation of Mn/Ni-PBA that delivered a capacity of approximately 100 mAh g^−1^ and an excellent rate performance, even at 100 C [185]. In particular, they reported that the activation in the first cycle at 4.8 V could significantly stabilize the structure, improving the cycling performance of the cathode. The presence of V, Co, and Ti increased the working potential of the PBA [186,187,188,189]. Takachi et al. used Na_x_Co[Fe(CN)_6_]_0.9_ as a SIB cathode, which delivered a capacity of approximately 135 mAh g^−1^ and demonstrated high-voltage redox couples of approximately 3.4 and 3.8 V versus Na/Na^+^ [190]. Baster reported that the replacement of Fe^2+^ with V^2+^ ions to form vanadium hexacyanoferrate (NaVHCF) exhibited a redox potential of ~2.3/3.6 V versus Na/Na^+^ [191]. Meanwhile, the presence of V–O binding in sodium vanadium hexacyanoferrate (NaVHCF) as a SIB cathode demonstrated only one redox potential [187]. Nguyen et al. reported that the presence of V–O passivated the high-spin Fe related to the low redox potential, supporting the structure of the low-spin Fe ion; therefore, NaVHCF provided a single redox potential of approximately 3.26/3.65 V versus Na/Na^+^ [192]. However, the capacity was only approximately 70–80 mAh g^−1^. 

Other MOFs have received considerable attention as anode materials owing to their new structures and stable redox potentials [193]. However, the application to SIBs as cathode materials remains limited, which may be owing to the fact that a high-voltage cathode is difficult to obtain, and the selection of large organic groups can lead to a decrease in conductivity [194]. In addition, the MOF structure can be used as a template to form oxide, nitride, carbide, and sulfide materials as anode materials in SIBs and for the design of cathode materials [195]. Li et al. used MIL-53(Al) to dope Al into Na_2_FePO_4_/C as a SIB cathode, where MIL-53(Al) was used as a template to form a porous structure with a carbon cover [196]. It delivered a high capacity of approximately 115 mAh g^−1^ [196] with stable cyclability.

## 3. Discussion

**Figure 9:**
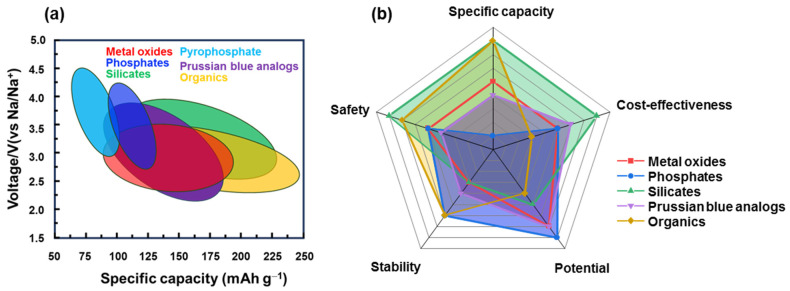
Comparison of SIB cathode materials’ (**a**) specific capacity and working potential; (**b**) specific capacity, cost-effectiveness, potential, stability, and safety issues.

LIBs have become popular in portable devices, vehicles, and energy storage systems for renewable energy. With the development of LIBs, a variety of cathode materials for SIBs have also recently evolved as listed in Table 1. Owing to the abundance of Na, SIBs are believed to be an ideal replacement for LIBs. As shown in Figure 9, each type of cathode material has its advantages and disadvantages. For instance, layered metal oxides have a high capacity and low cost but are sensitive to moisture and structural degradation. Prussian blue is more stable but the effect of water molecules in the structure affects its performance. Organic cathode materials have a good flexibility and stable redox potential but their lower conductivity, thermal stability, and dissolvability in the electrolyte should be resolved. Therefore, the advantages and disadvantages of each practical condition should be carefully considered. To improve their performance, the approach methods were also varied for each type of material. Due to an instability in structure of layered metal oxide cathodes, they were fast degraded during cycling. To stabilize structural stability, inactive metals such as V, Mg, Zn, and Ca can be doped to the lattice, or anions like F can be added [197,198]. Considering a tunnel metal oxide, control of the tunnel size optimizes its capacity. Meanwhile, for polyanionic compounds such as NASICON or other phosphate-based compounds, defect engineering can be considered, including metal- and F-doping methods [199]. Silicate compounds are low-cost and eco-friendly metal sources, and their high capacity needs to improve the structural stability before commercialization [154]. The surfaces of inorganic compounds can be passivated using a carbon-coating method that not only enhances their conductivity but also protects against the effects of humidity or expansion during the insertion of Na ions. The stability of Prussian blue and other organometallic compounds can be enhanced by using a host material such as Ni foam or a porous carbon skeleton [200]. Organic materials can be designed to have a good structure to enhance capacity and conductivity but they remain in the activation group with C=O, C=C, or C=N. Sulfurization and other cross-linking methods can also be considered to yield better combinations [201]. In addition, the use of additives in the electrolyte is another approach to enhance stability, in which the solid electrolyte interface from cycling can be used as a protective layer [202]. Along with the development of electrode materials and electrolytes, SIBs have been commercialized with layered oxides, polyanions, and Prussian blue types [32]. These materials are simple to manufacture (hydrothermal, co-precipitation method, etc.) and inexpensive, and they mainly use Mn and Fe metals and add Ni, Zn, or Mg, to increase stability, and conductive carbon is introduced for air stability and structural protection. Organic materials with low thermal stability and conductivity are utilized for some specific purposes that require biocompatible and/or specified applications. Therefore, it is considered that most of the developed materials have the potential to be commercialized if SIBs can solve current issues such as cost-effectiveness, high capacity, high stability, and high rate performance.

In summary, this review reveals the current developments in SIB cathode materials, such as layered metal oxides, tunnel metal oxides, phosphate-based compounds, organic compounds, Prussian blue analogs, and organometallic compounds. Many methods including anion/cation doping, composition, defect engineering, and structural design of SIB cathodes demonstrate their significant development to be comparable with LIBs. However, the development of SIBs can be further investigated to optimize the process or engineer the structure and design of cathode materials with high capacity, high voltage potential, and long life.

## Figures and Tables

**Table 1:** Summary of SIB cathode materials.

Materials	Redox Couple	Working Voltage (V)	Current Density	Specific Capacity (mAh g^−1^)	Ref.
LAYERED OXIDES					
Na_0.7_CoO_2_	Co^3+^/Co^4+^	2.0–3.5	0.08 C	70.4	[48]
Na_x_CoO_2_	Co^3+^/Co^4+^	2.0–4.0	0.1 C	121	[50]
Na_0.44_Mn_1−x_Co_x_O_2_	Co^3+^/Co^4+^ Mn^3+^/Mn^4+^	2.0–4.2	0.1 C	220	[103]
NaFeO_2_	Fe^3+^/Fe^4+^	2.0–3.6	0.15 C	101	[86]
NaTi_0.5_Ni_0.5_O_2_	Ni^2+^/Ni^3+^ Ti^3+^/Ti^4+^	2.0–3.8	0.23 C	130	[203]
NaFe_0.5_Ni_0.5_O_2_	Ni^3+^/Ni^4+^ Fe^2+^/Fe^3+^	2.0–3.8	0.24 C	125	[203]
NaNiO_2_	Ni^2+^/Ni^3+^	1.25–3.75	0.02 C	120	[49]
Na_2_NiO_2_	Ni^3+^/Ni^4+^	2.0–3.6	0.05 C	89	[51]
NaCrO_2_:Na_2_NiO_2_	Ni^3+^/Ni^4+^ Cr^2+^/Cr^3+^	2.0–3.6	0.05 C	107	[51]
NaMnO_2_	Mn^2+^/Mn^3+^	2–3.8	0.05 C	185	[55]
β-NaMnO_2_	Mn^3+^/Mn^4+^	2–4.2	0.05 C	190	[56]
Na_0.7_MnO_2_	Mn^3+^/Mn^4+^	2.0–4.5	0.25 C	163	[43]
Na_0.67_Mn_0.85_Al_0.15_O_2_	Mn^3+^/Mn^4+^	2.0–4.0	0.05 C	104	[63]
Na_2_/_3_Ni_1_/_3_Mn_2_/_3_O_2_	Mn^3+^/Mn^4+^ Ni^3+^/Ni^4+^	2.0–4.0	0.1 C	89	[67]
Na_0.7_Mn_0.93_Li_0.07_O_2_	Mn^3+^/Mn^4+^	2.0–3.8	0.4 C	183	[60]
Na_0.95_Li_0.15_(Ni_0.15_Mn_0.55_Co_0.1_)O_2_	Mn^3+^/Mn^4+^ Ni^3+^/Ni^4+^	2.0–4.2	0.05 C	200	[72]
Na_0.80_[Li_0.12_Ni_0.22_Mn_0.66_]O_2_	Mn^3+^/Mn^4+^ Ni^3+^/Ni^4+^	2.0–4.0	0.1 C	120	[74]
Na_0.67_Ni_0.33_Mn_0.67_Y_0.02_O_2_	Mn^3+^/Mn^4+^ Ni^3+^/Ni^4+^	2.0–4.5	0.05 C	137	[77]
TUNNEL OXIDES					
Na_0.44_MnO_2_	Mn^3+^/Mn^4+^	2.0–4.0	10 C	96	[87]
Na_0.44_Mn_0.98_Zr_0.02_O_2_	Mn^3+^/Mn^4+^	2.0–3.8	0.1 C	117	[101]
Na_0.44_MnO_2_⋅Na_2_Mn_3_O_7_	Mn^3+^/Mn^4+^	1.5–4.6	1.37 C	145	[102]
Na_2/3_Mn_0.95_Mg_0.05_O_2_	Mn^3+^/Mn^4+^	1.5–4.0	7 C	140	[78]
POLYANION COMPOUNDS					
NaFePO_4_	Fe^2+^/Fe^3+^	2.2–4.0	0.07 C	142	[111]
Na_3_V_2_(PO_4_)_3_	V^3+^/V^4+^ V^2+^/V^3+^	2.0–4.6	0.1 C	117.6	[123]
Na_3_-xKxV_2_(PO_4_)_3_/C	V^3+^/V^4+^ V^2+^/V^3+^	2.5–3.8	0.2 C	~110	[125]
Na_3_V_1.93_Mg_0.07_(PO_4_)_3_/C	V^3+^/V^4+^ V^2+^/V^3+^	2.3–4.6	0.1 C	113.5	[127]
Na_3.1_V_2_(PO_4_)_2.9_(SiO_4_)_0.1_/C	V^3+^/V^4+^ V^2+^/V^3+^	2.5–4.0	0.1 C	112	[126]
Na_3_V_2_(PO_4_)_2_F_3_	V^3+^/V^4+^ V^2+^/V^3+^	2.0–4.6	0.1 C	117	[129]
PYROPHOSPHATES					
Na_3.32_Fe_2.34_(P_2_O_7_)_2_	Fe^2+^/Fe^3+^	1.7–4.0	0.05 C	117.6	[138]
Na_2_FeP_2_O_7_	Fe^2+^/Fe^3+^	2.0–4.5	0.05 C	90	[141]
Na_2_CoP_2_O_7_	Co^2+^/Co^3+^	1.6–4.5	0.05 C	80	[134]
Na_2_MnP_2_O_7_	Mn^3+^/Mn^4+^	2.0–4.45	0.05 C	80	[135]
Na_4_NiP_2_O_7_F_2_	Ni^2+^/Ni^3+^/Ni^4+^	3.0–5.5	0.01 C	50	[145]
Na_2_Mn_3_-xFex(P_2_O_7_)_2_	Fe^2+^/Fe^3+^ Mn^3+^/Mn^4+^	1.5–4.5	0.058 C	86.8	[142]
Na_4_Fe_3_(PO_4_)_2_P_2_O_7_/C	Fe^2+^/Fe^3+^	1.5–4.2	0.2 C	128.5	[144]
Silicates					
Na_2_FeSiO_4_	Fe^2+^/Fe^3+^/Fe^4+^	1.5–4.5	0.1 C	271	[157]
Na_2_MnSiO_4_	Mn^3+^/Mn^4+^	2.0–4.3	0.1 C	210	[146]
Na_2_CoSiO_4_	Co^2+^/Co^3+^	1.5–4.0	0.05 C	112	[156]
Na_2_CoSiO_4_/CNT	Co^2+^/Co^3+^	1.5–4.0	0.05 C	125	[156]
Organic materials					
Na_4_C_8_H_2_O_6_	Na_2_C_8_H_2_O_6_/Na_4_C_8_H_2_O_6_	1.6–2.8	0.1 C	180	[159]
PTCDA based Polyimides	Na_2_PI/Na_4_PI	1.5–3.5	0.2 C	112	[160]
NTCDA based Polyimides	Na_2_PI/Na_4_PI	1.5–3.5	0.2 C	125	[160]
PMDA based Polyimides	Na_2_PI/Na_4_PI	1.5–3.5	0.2 C	133	[160]
Poly(diphenylaminesulfonic acid sodium) (PDS)	-	2.5–4.2	0.5 C	100	[162]
Bipolar porous organic electrode	-	1.3–4.1	0.05 C	200	[165]
Prussian blue analogs					
Na_4_Fe(CN)_6_/NaCl	Fe^2+^/Fe^3+^	1.5–3.9	0.93 C	75	[177]
Na_4_Fe(CN)_6_/C	Fe^2+^/Fe^3+^	2.0–4.0	0.1 C	90	[175]
Na_2_MnFe(CN)_6_	Mn^2+^/Mn^3+^	2.0–4.0	0.1 C	150	[183]
NaxMn_0.5_Ni_0.5_Fe(CN)_6_	Mn^2+^/Mn^3+^	2.0–4.0	0.1 C	100	[185]
NaxCo[Fe(CN)_6_]_0.9_	Fe^2+^/Fe^3+^ Co^2+^/Co^3+^	2.0–4.0	0.6 C	135	[185]
NaxVOFe(CN)_6_	Fe^2+^/Fe^3+^	2.0–4.0	0.25 C	65	[187]

## Data Availability

Not applicable.
